# Antimicrobial prophylaxis with teicoplanin plus gentamicin in primary total joint arthroplasty

**DOI:** 10.5194/jbji-8-219-2023

**Published:** 2023-10-30

**Authors:** Tariq Azamgarhi, Craig Gerrand, John A. Skinner, Alexander Sell, Robert A. McCulloch, Simon Warren

**Affiliations:** 1 Pharmacy Department, Royal National Orthopaedic Hospital NHS Trust, Stanmore, HA7 4LP, UK; 2 Division of Orthopaedic Oncology, Royal National Orthopaedic Hospital NHS Trust, Stanmore, HA7 4LP, UK; 3 Research and Innovation Centre, Royal National Orthopaedic Hospital NHS Trust, Stanmore, HA7 4LP, UK; 4 Division of Surgery and Interventional Science, University College London, London, WC1E 6BT, UK; 5 Department of Anaesthesia, Royal National Orthopaedic Hospital NHS Trust, Stanmore, HA7 4LP, UK; 6 Joint Reconstruction Unit, Royal National Orthopaedic Hospital NHS Trust, Stanmore, HA7 4LP, UK; 7 Bone Infection Unit, Royal National Orthopaedic Hospital NHS Trust, Stanmore, HA7 4LP, UK; 8 The Royal Free Hospital NHS Foundation Trust, Hampstead, London, NW3 2QG, UK

## Abstract

**Objectives**: To compare prosthetic joint infection (PJI) and acute kidney injury (AKI) rates among cohorts before and after changing our hospital's antimicrobial prophylactic regimen from cefuroxime to teicoplanin plus gentamicin. **Methods**: We retrospectively studied all patients undergoing primary total joint arthroplasty at our hospital 18 months pre- and post-implementation of the change in practice. All deep infections identified during follow-up were assessed against the European Bone and Joint Infection Society (EBJIS) definitions for PJI. Survival analysis using Cox regression was employed to adjust for differences in baseline characteristics and compare the risk of PJI between the groups. AKIs were identified using pathology records and categorized according to the KDIGO (Kidney Disease – Improving Global Outcomes) criteria. AKI rates were calculated for the pre- and post-intervention periods. **Results**: Of 1994 evaluable patients, 1114 (55.9 %) received cefuroxime only (pre-intervention group) and 880 (44.1 %) patients received teicoplanin plus gentamicin (post-intervention group). The overall rate of PJI in our study was 1.50 % (30 of 1994), with a lower PJI rate in the post-intervention group (0.57 %; 5 of 880) compared with the pre-intervention group (2.24 %; 25 of 1114). A corresponding risk reduction for PJI of 75.2 % (95 % CI of 35.2–90.5; p=0 .004) was seen in the post-intervention group, which was most pronounced for early-onset and delayed infections due to coagulase-negative staphylococci (CoNS) and cefuroxime-resistant Enterobacteriaceae. Significantly higher AKI rates were seen in the post-intervention group; however, 84 % of cases (32 of 38) were stage 1, and there were no differences in the rate of stage-2 or -3 AKI. **Conclusions**: Teicoplanin plus gentamicin was associated with a significant reduction in PJI rates compared with cefuroxime. Increases in stage-1 AKI were seen with teicoplanin plus gentamicin.

## 1 Introduction

Prosthetic joint infection is a devastating complication of total joint arthroplasty (TJA). Antimicrobial prophylaxis is one of several measures recommended to reduce infection. No regimen is known to be superior to another; therefore, various regimens are in use (Aujla et al., 2013). Cephalosporins are the most widely used and studied antibiotics in orthopaedic surgery; however, in the UK, many hospitals have moved away from cephalosporins because of concerns about *Clostridioides difficile* infection (CDI) (Aujla et al., 2013). In September 2013, we changed the first-line antibiotic regimen for surgical prophylaxis in TJA from cefuroxime (1.5 g every 8 h for three doses with the first dose at induction) to single doses of teicoplanin (10 mg kg^-1^) plus gentamicin (5 mg kg^-1^) at induction. No dose adjustments were recommended for patients with renal impairment.

The decision to use teicoplanin was based on high susceptibility amongst commonly infecting Gram-positive organisms including *Staphylococcus aureus* and coagulase-negative staphylococci. Gentamicin was added to provide Gram-negative cover. Both have a long half-life, avoiding re-dosing even for lengthy procedures.

This study aims to assess the impact of implementing this change in practice. The specific objective was to calculate the incidence of deep infection and acute kidney injury (AKI) before and after the change.

## 2 Methods

This was a retrospective review of clinical records, comparing cohorts before and after the change in antibiotic regimen at our specialist orthopaedic hospital. The pre-intervention group included all patients undergoing total hip or knee arthroplasty in the 18 months before the change in prophylaxis (i.e. from January 2012 to September 2013). The post-intervention group included all total hip arthroplasty (THA) or total knee arthroplasty (TKA) patients in the 18 months after the change (i.e. from October 2013 to March 2015).

All primary hip and knee arthroplasty patients were identified using a prospectively maintained hip and knee arthroplasty register employed for monitoring surgical site infection rates by the hospital's infection prevention and control (IPC) team. Patient information was gathered from electronic notes and pathology records including the following: demographics (age and sex); comorbidities, including obesity, hypertension, diabetes mellitus, malignancy, liver disease, lung disease or chronic renal failure; preoperative performance status, measured by use of the American Association of Anaesthesiology (ASA) classification; laterality; type of implant (TKA or THA); duration of surgery; preoperative and postoperative (day-7) haemoglobin and creatinine values; and the number of red blood cell transfusions.

At our hospital, antimicrobial prophylaxis is prescribed but not dispensed individually for patients and is available as a stock medicine in theatres where the anaesthetic team prepare doses on a case-by-case basis. Only the current recommended protocol choice of antimicrobial is stocked in theatres. Patients with a penicillin allergy or those who are known to be colonized with methicillin-resistant *Staphylococcus aureus* (MRSA) have alternative antibiotic prophylaxis prescribed and issued by the pharmacy department on a named-patient basis. Therefore, pharmacy records were used to identify patients in the pre-intervention group who received alternatives to cefuroxime due to penicillin allergy.

All surgery was performed in an operating room with laminar airflow. Throughout the study, there were no variations in the preoperative washing protocol, skin preparation method, hand hygiene solutions used, type of surgical equipment sterilization, operating theatres or postoperative wound management protocols. Screening for MRSA carriers was performed, and decolonization was provided to all carriers identified during the study period.

### 2.1 Prosthetic joint infection

Patients were routinely followed up within 6 weeks, at 3 months and at least 12 months. Patients diagnosed with an infection were identified using pathology records, pharmacy issues of inpatient and outpatient antimicrobial supply, clinical infection service records, surgical site infection surveillance registry, and hospital records of readmission. All suspected prosthetic joint infections (PJIs) were assessed and graded as likely or confirmed PJI according to recently published diagnostic criteria (McNally et al., 2021).

The clinical features of infection at presentation were collected. Infections were classified as early if a diagnosis of infection was made < 3 months after surgery, delayed if diagnosis of infection was made 3 to 12 months after surgery, or late if diagnosis of infection was made 12 to 24 months after the index procedure.

All patients with infection returned to theatres, and deep samples of synovial fluid and periprosthetic tissue were sent for microbiological analysis. Organisms identified were grouped into *Staphylococcus aureus*, coagulase-negative staphylococci (CoNS), *Streptococcus* spp., *Enterococcus* spp., cefuroxime-sensitive or cefuroxime-resistant Enterobacteriaceae, *Pseudomonas aeruginosa*, polymicrobial infection or no cultured organism (NCO).

### 2.2 Acute kidney injury 

Preoperative serum creatinine was defined as the value up to 3 months before surgery, whereas postoperative creatinine was defined as the level within 7 d after surgery. The change in pre-postoperative creatinine was calculated and categorized as stage-0 to stage-3 AKI according to the KDIGO (Kidney Disease – Improving Global Outcomes) criteria for acute kidney injury (Khwaja, 2012).

## 3 Statistical analysis

Data were summarized using descriptive statistics. Continuous variables were expressed as the median and interquartile range (IQR) and were compared using a Student t test or a Mann–Whitney U test. Categorical data on baseline characteristics were compared using a two-sided Pearson $\chi^{2}$ test or a Fisher exact test.

We applied survival analysis to investigate the impact of different prophylactic regimens on PJI. The outcome variable was time to likely or confirmed PJI according to published criteria, constructed as the time between index surgery and the diagnosis of PJI. Patients lost to follow-up or followed up without PJI at 2 years were censored. The Kaplan–Meier method was used to plot cumulative hazards for the cefuroxime and teicoplanin plus gentamicin groups.

Cox regression was used for multivariate analysis. Initially, a simple “unadjusted” comparison was made between groups. Secondly, a multivariate analysis was performed, adjusting for baseline characteristics found to vary significantly between the pre-intervention and post-intervention groups was performed. Statistical significance was defined as a two-tailed p value of < 0.05. Statistical analysis was performed using SPSS version 25.0 (IBM, Chicago, IL).

The study was approved by the Research and Innovation Committee. Due to anonymized data, a waiver of consent for clinical data collection was granted.

## 4 Results

In total, 2059 patients underwent TKA or THA during the study period, of which 65 (3.2 %) were excluded in the pre-implementation group as they did not receive cefuroxime because of a penicillin allergy or known MRSA colonization. After exclusions, 1994 patients were analysed: 1114 (55.9 %) received cefuroxime only (pre-intervention group) and 880 (44.1 %) patients received teicoplanin plus gentamicin (post-intervention group).

The baseline characteristics of the pre- and post-intervention groups were compared (Table 1). There was a higher proportion of female patients (67 % vs. 60 %, p=0 .006) and more cemented arthroplasties (69 % vs. 64 %, p=0 .0254) in the pre-intervention group. There were no significant differences in body mass index (BMI), ASA grade, comorbidities, side, length of procedure, anatomic site (hip or knee) or haemoglobin (Hb) values.

**Table 1:** Baseline characteristics of pre- and post-intervention groups.

Characteristics	Pre–intervention	Post-intervention	p value
	(cefuroxime)	(teicoplanin plus gentamicin)	
	N=1114	N=880	
Median (IQR) age (year)	66 (54–74)	66 (56–74)	0.713
No. (%) of patients by sex			
Female	742 (66.6)	534 (60.7)	**0.006**
Male	372 (33.4)	346 (39.4)	
No. (%) of patients with a BMI within the following ranges:			
< 30 kg m^-2^	913 (82.1)	731 (83.1)	0.517
30– < 35 kg m^-2^	171 (15.4)	123 (14.0)	0.391
≥35 kg m^-2^	30 (2.7)	26 (3.0)	0.726
No. (%) of patients with the following ASA grades:			
ASA 1 or 2	942 (84.6)	741 (84.2)	0.620
ASA 3 or 4	172 (15.4)	139 (15.8)	
No. (%) of patients with the following comorbidities:			
Hypertension	490 (44.0)	393 (44.7)	0.764
Diabetes mellitus	124 (11.1)	97 (11.0)	0.939
Malignancy	34 (3.1)	19 (2.2)	0.218
Liver disease	9 (0.8)	13 (1.5)	0.155
Lung disease	180 (16.2)	181 (20.6)	0.826
Chronic kidney disease	26 (2.3)	25 (2.8)	0.476
Rheumatoid arthritis	50 (4.5)	36 (4.1)	0.664
No. (%) of patients with the following laterality:			
Left	506 (45.4)	419 (47.6)	0.330
Right	608 (54.5)	461 (52.4)	
Median (IQR) surgery duration (mins)	91 (75–113)	94 (75–110)	0.923
No. (%) of patients with a duration of surgery of ≥ 90 min	567 (50.9)	430 (48.9)	0.764
No. (%) of patients with arthroplasty at the following sites:			
Knee	619 (55.6)	505 (57.4)	0.416
Hip	495 (44.4)	375 (42.6)	
No. (%) of patients with a cemented prosthesis:			
Cemented	763 (68.5)	561 (63.7)	**0.026**
Uncemented	351 (31.5)	319 (36.3)	
Median (IQR) Hb value (g dL^-1^)			
Preoperative	13.3 (12.4–14.3)	13.5 (12.6–14.3)	0.404
Postoperative	10.7 (10.7–11.7)	9.9 (11.0–11.9)	0.135
No. (%) of patients with the following red blood cell transfusion amounts:			
1 unit	15 (1.3)	15 (1.7)	
2 units	86 (7.7)	54 (6.1)	0.265
≥3 units	28 (2.5)	14 (1.6)	

Bold values denote statistical significance at the p<0.05 level.

**Figure 1:**
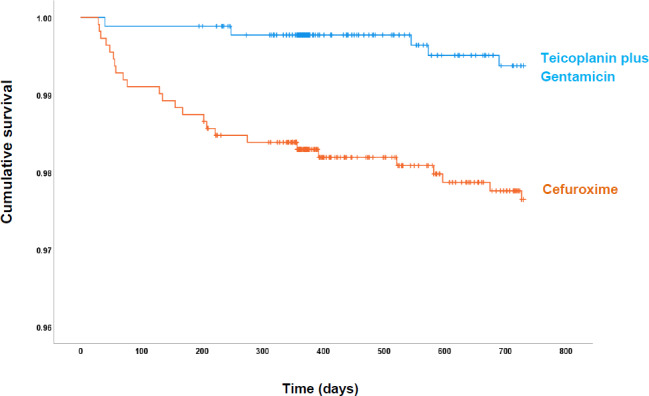
Cumulative probability of being free of PJI within 730 d of follow-up for each antibiotic prophylactic regimen: cefuroxime (orange) and teicoplanin plus gentamicin (blue).

**Figure 2:**
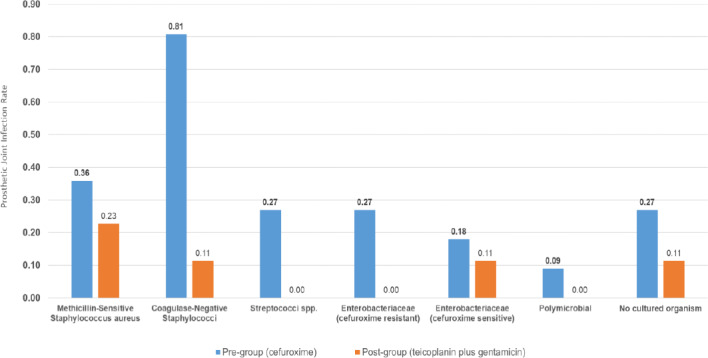
PJI rate by microorganism group according to the antibiotic prophylaxis administered.

A total of 30 infections were identified within 2 years of implantation: 25 in the pre-intervention group (cefuroxime) and 5 in the post-intervention group (teicoplanin plus gentamicin) (Fig. 1, Table 2). The microbiological profiles of PJI in each group are shown in Fig. 2.

**Table 2:** Comparison of time to PJI in the cefuroxime and teicoplanin plus gentamicin groups.

Analysis	Cefuroxime	Teicoplanin plus	Hazard ratio	p value
	n/N	gentamicin n/N	(95 % CI)^*^	
Unadjusted	25/1114	5/880	0.240 (0.092–0.629)	0.004
Adjusted	–	–	0.248 (0.095–0.648)	0.004

Cox hazard ratios and 95 % confidence intervals are reported with corresponding p values. The p values were estimated by the Wald test. Statistical significance was set at p<0 .05, and two-sided p values were used. Analyses were adjusted for sex and cement use. ^*^ Expressed as hazard of PJI in the post-intervention group (teicoplanin plus gentamicin) relative to the pre-intervention group (cefuroxime).

The overall rate of PJI in our study was 1.50 % (30 of 1994), with a lower PJI rate in the post-intervention group (0.57 %; 5 of 880) compared with the pre-intervention group (2.24 %; 25 of 1114) (Fig. 1). Most infections in the pre-intervention group presented during the first 90 d after surgery. However, the survival curves continued to diverge up to 250 d follow-up. Overall, the post-intervention group had a statistically significant risk reduction (hazard ratio, HR, 0.25: 95 % CI 0.09–0.64; p= 0.004) for PJI in the adjusted analysis (Table 2).

The clinical features of patients with infection are shown in Table 4. Of the 25 patients with infections in the pre-intervention group, 17 (68.0 %) were either early or delayed, and 8 (32.0 %) were late infections. Early and delayed infections were most commonly due to CoNS (47.1 %), followed by cefuroxime-resistant Enterobacteriaceae (17.6 %), methicillin-susceptible *Staphylococcus aureus* (MSSA; 17.6 %), NCO (11.8 %) and *Streptococci* spp. (5.9 %). Late infections (32.0 %) were more often due to *Streptococci* spp., cefuroxime-sensitive Enterobacteriaceae or MSSA.

**Table 3:** Incidence of perioperative acute kidney injury (AKI) in the cefuroxime and teicoplanin plus gentamicin groups according to the KDIGO criteria. n/a – not applicable

AKI grade	Cefuroxime	Teicoplanin plus	p value
		gentamicin	
No. (%) of patients with any AKI	9 (0.8)	29 (3.0)	0.001
No. (%) of patients with AKI 1	6 (0.5)	26 (3.0)	0.001
No. (%) of patients with AKI 2	3 (0.3)	3 (0.3)	0.772
No. (%) of patients with AKI 3	0	0	n/a

**Table 4:** Clinical details of PJIs in pre-intervention (PRE) and post-intervention (POST) groups.

Group	Time	Chronicity	EBJIS	Organism	AmpC or	Microbiology	Antimicrobial			Surgery for
			criteria	group	ESBL		susceptibilities			infection
							Teicoplanin	Gentamicin	Cefuroxime	
PRE	29	Early	Definite	Enterobacteriaceae (cefuroxime resistant)	ESBL	*Escherichia coli*	NA	S	R	Washout and debridement
PRE	33	Early	Definite	CoNS	NA	*Staphylococcus epidermidis*	R	R	R	Washout and debridement
PRE	42	Early	Definite	CoNS	NA	*Staphylococcus epidermidis*	S	R	R	Exploration and washout
PRE	45	Early	Likely	NCO	NA	No cultured organism	NA	NA	NA	DAIR
PRE	49	Early (haematogenous)	Definite	*Staphylococcus aureus*	NA	*Staphylococcus aureus*	S	S	S	Washout and debridement
PRE	54	Early	Definite	CoNS	NA	*Staphylococcus simulans*	S	S	S	DAIR
PRE	58	Early	Definite	NCO	NA	NA	S	S	R	DAIR
PRE	70	Early	Definite	CoNS	NA	*Staphylococcus epidermidis*	S	R	R	DAIR
PRE	77	Early	Definite	*Staphylococcus aureus*	NA	*Staphylococcus aureus*	S	S	S	DAIR
PRE	131	Delayed (haematogenous)	Definite	*Streptococcus* spp.	NA	Group-B *Streptococcus*	S	S	S	Washout and debridement
PRE	156	Delayed	Definite	*Staphylococcus aureus*	NA	*Staphylococcus aureus*	S	S	S	Washout and debridement
PRE	169	Delayed	Definite	CoNS	NA	*Staphylococcus epidermidis*	S	R	R	Washout and debridement
PRE	208	Delayed	Definite	Enterobacteriaceae (cefuroxime resistant)	AmpC	*Morganella morganii*	R	S	R	Two-stage revision
PRE	210	Delayed	Definite	Enterobacteriaceae (cefuroxime resistant)	ESBL	*Escherichia coli* (ESBL)	R	S	R	Two-stage revision
PRE	222	Delayed	Definite	CoNS	NA	*Staphylococcus lugdunensis*	S	R	S	Debridement
PRE	276	Delayed	Definite	CoNS	NA	*Staphylococcus epidermidis*	S	S	R	Two-stage revision
PRE	358	Delayed	Definite	CoNS	NA	*Staphylococcus lugdunensis*	S	S	S	Washout and debridement
PRE	392	Late	Definite	Enterobacteriaceae (cefuroxime sensitive)	No	*Citrobacter koseri*	NA	S	S	Single-stage revision
PRE	521	Late	Definite	CoNS	NA	*Staphylococcus epidermidis*	S	R	R	Two-stage revision
PRE	582	Late	Definite	Polymicrobial	No	*Staphylococcus aureus* and Enterobacter sp.	NA	NA	NA (Enterobacter sensitive)	Washout and debridement
PRE	597	Late (haematogenous)	Likely	NCO	NA	No cultured organism	NA	NA	NA	Two-stage revision
PRE	707	Late	Definite	*Streptococcus* spp.	NA	Group-B *Streptococcus*	S	S	S	Washout
PRE	729	Late (haematogenous)	Definite	*Staphylococcus aureus*	NA	*Staphylococcus aureus*	S	S	S	Two-stage revision
PRE	730	Late	Likely	Enterobacteriaceae (cefuroxime sensitive)	No	*Proteus mirabilis*	NA	S	S	Two-stage revision

**Table 4:** Continued.

Group	Time	Chronicity	EBJIS	Organism	AmpC or	Microbiology	Antimicrobial			Surgery for
			criteria	group	ESBL		susceptibilities			infection
							Teicoplanin	Gentamicin	Cefuroxime	
PRE	730	Late (haematogenous)	Definite	*Streptococcus* spp.	NA	*Streptococcus pneumoniae*	S	S	S	Washout and debridement
POST	40	Early	Likely	NCO	NA	No cultured organism	NA	NA	NA	Washout and debridement
POST	248	Delayed	Definite	*Staphylococcus aureus*	NA	*Staphylococcus aureus*	S	S	R	Two-stage revision
POST	554	Late	Definite	CoNS	NA	*Staphylococcus epidermidis*	I	R	R	DAIR
POST	574	Late (haematogenous)	Likely	Enterobacteriaceae (cefuroxime sensitive)	No	*Escherichia coli*	NA	S	S	DAIR
POST	690	Late	Definite	*Staphylococcus aureus*	NA	*Staphylococcus aureus*	S	S	S	DAIR

The notation used in table is as follows: extended-spectrum β -lactamases – ESBL; AmpC β -lactamases – AmpC; NA – not available; R – resistant; S – sensitive; I – intermediate; CoNS – coagulase-negative staphylococci; NCO – no cultured organisms; DAIR – debridement, antibiotics and implant retention; and EBJIS – European Bone and Joint Infection Society.

In the post-intervention group, two (40 %) were early or delayed, one was MSSA and one was NCO, while three (60 %) were late infections. Of the three late infections, one was due to MSSA, one was due to CoNS and one was due to cefuroxime-sensitive *Escherichia coli*.

The overall AKI rates in the study were 1.9 % (38 of 1994): 0.8 % (9 of 1114) in the pre-intervention group and 3.3 % (29 of 880) in the post-intervention group (Table 3). A total of 84 % (32 of 38) of all AKIs were stage 1, with a statistically higher rate in the post-intervention group ( p=0.001 ). There was no difference in the rate of stage-2 AKI between the groups, and no patients developed stage-3 AKI in the study. No patients required renal replacement therapy for the treatment of AKI.

## 5 Discussion

This large single-centre pre- and post-intervention study was designed to evaluate the effect of changing antimicrobial prophylaxis from cefuroxime to teicoplanin plus gentamicin on the occurrence of PJI and AKI. The study was conducted over a period that avoided significant changes in IPC policy, minimizing the potential impact on infection rates.

Pre- and post-intervention groups were compared for significant differences in patient or surgical factors. The only significant differences were a higher proportion of males and the use of uncemented prostheses in the post-intervention group; therefore, we adjusted for these differences to enable comparison between the groups.

The change in prophylaxis was associated with a reduction in deep PJI from 2.24 % to 0.57 % at 2 years. We evaluated the rate of deep infection for two reasons: firstly, deep infections are the most important, due to their high clinical impact, and are costly to treat; secondly, it can be challenging to identify superficial and incisional infections retrospectively. We used multiple methods to identify deep infections, so we are confident that none were missed. Infections were categorized by applying the same published definitions to both groups.

Survival analysis showed that the post-intervention group had a 75.2 % (95 % CI 35.2–90.5; p=0 .004) risk reduction for PJI, which was most pronounced for early-onset and delayed infections due to CoNS and cefuroxime-resistant Enterobacteriaceae. These infections are commonly acquired during surgery and are preventable by multiple measures, including antibiotic prophylaxis, theatre IPC practice and postoperative wound management. The change in antibiotic prophylaxis is the most likely reason for the lower infection risk in the post-intervention group for several reasons. Firstly, there were no significant changes in the other infection prevention measures during the study. Secondly, cefuroxime selected specific organism groups due to its poor activity against CoNS and Enterobacteriaceae. Thirdly, the infecting organisms selected out by cefuroxime would have been covered with teicoplanin and gentamicin. Therefore, we postulate that the change in antibiotic prophylaxis is the most likely reason for the lower infection risk in the post-intervention group, as there were no significant changes to the other infection prevention measures during the study.

We saw a reduction in early and delayed infections due to CoNS or cefuroxime-resistant Enterobacteriaceae. Cefuroxime has a lack of or poor activity against these organisms, which are better covered with teicoplanin and gentamicin. This provides further evidence of an effect from the change in prophylaxis.

Prospective trials of antibiotic prophylaxis have not found any regimen to reduce risk compared to another. However, these trials only compared single-antibiotic regimens with minor differences in antimicrobial spectrum (Siddiqi et al., 2019). They were also conducted 3–4 decades ago when antimicrobial resistance trends differed (Siddiqi et al., 2019). More recently, retrospective studies have compared β -lactams alone or in addition to a glycopeptide (Branch-Elliman et al., 2017, 2019; Burger et al., 2018; Capdevila et al., 2016; Liu et al., 2014; Sewick et al., 2012; Tornero et al., 2015). Tornero et al. (2015) suspected the underdosing of cefuroxime to be a potential cause of the higher rate of PJIs with cefuroxime alone compared with cefuroxime with the addition of teicoplanin (3.51 % vs. 1.26 %, p=0 .002) in hip and knee arthroplasty. The addition of teicoplanin was associated with significantly lower rates of *Staphylococcus aureus* infection. Secondary analysis of the cefuroxime group found a higher infection rate in obese patients compared with the group overall (4.5 % vs. 3.5 %). In our study, 24 of 25 (96 %) infections in the cefuroxime group occurred in obese patients, whereas 2 of 5 (40 %) occurred in obese individuals in the teicoplanin plus gentamicin group. The median duration of surgery in our study was 91–94 min, which is shorter than the half-life of cefuroxime; thus, if underdosing is an issue in obese patients, it is more likely related to the initial dose (Asín-Prieto et al., 2015).

Concerns were raised about aminoglycoside toxicity when considering an alternative to cefuroxime. AKI rates were higher in the post-intervention group; however, a significant difference occurred only with stage-1 AKI (3.0 % vs. 0.5 %; p=0 .001). All patients were managed conservatively, and none required renal replacement therapy. A pre- and post-intervention study comparing AKI rates with cefuroxime and flucloxacillin plus gentamicin found a higher rate with the combined regimen (Bell et al., 2014). However, the difference was only seen with stage-1 AKI, which is consistent with our study. It is impossible to attribute the AKI risk to a particular antibiotic or account for baseline risk, but there were similar rates of chronic disease, hypertension and diabetes between the groups.

Anaphylaxis is an important consideration, as it increases mortality in the perioperative setting. The 6th National Audit Project (NAP6) estimated the incidence of anaphylaxis for different prophylactic antibiotics. The incidence per 100 000 was highest for teicoplanin at 16.4, followed by cefuroxime at 0.9 and gentamicin at 0.6 (Cook et al., 2018). Of particular concern are the findings of an observational study at our hospital. We estimated the actual rate to be 3 times higher than NAP6, ranging between 1:2088 and 1:1655 , possibly due to the underreporting of anaphylaxis during NAP6 (Azamgarhi et al., 2018). We recommended that teicoplanin should be administered over 30 min so that infusion-related reactions are unlikely.

All decisions require a balance of risk and benefits. If we were to consider 10 000 TJA surgeries where teicoplanin plus gentamicin was used in preference to cefuroxime, there would be 125 fewer PJIs; however, based on the higher rate of anaphylaxis for teicoplanin and NAP6 data for cefuroxime and gentamicin, 220 more stage-1 AKIs and 5 more cases of life-threatening anaphylaxis would occur.

The nature of the study design compared two patient groups over two consecutive 18-month periods. The pre- and post-implementation groups were similar; however, there may have been some minor differences in patient comorbidities and the organisms identified that could have contributed to the overall outcome for each group. Although there were no significant changes in the hospital's IPC procedures or system processes during the study, it is impossible to eliminate the historical effects on patients treated between consecutive periods.  The incidence of vancomycin-resistant enterococci in our hospital was too low to assess the impact of moving to teicoplanin as routine prophylaxis and requires further study. While acknowledging these limitations, these findings provide helpful information for hospitals considering the risk vs. benefits of different antimicrobial prophylactic regimens.

Hospitals need to consider whether the benefits of teicoplanin plus gentamicin outweigh the harms, such as anaphylaxis. Prospective multicentre studies are required to evaluate extended-spectrum prophylaxis on efficacy, safety and antimicrobial resistance.

## 6 Conclusions

In total joint arthroplasty, changing to prophylaxis with teicoplanin plus gentamicin was associated with a significant reduction in PJI rates compared with cefuroxime. This was primarily due to a reduction in early and delayed infections caused by caused by CoNS and cefuroxime-resistant Enterobacteriaceae. Increases in stage-1 AKI were seen with teicoplanin plus gentamicin.

## Data Availability

The datasets used and/or analysed during the current study are available from the corresponding author on reasonable request.
